# Rhodocetin-αβ selectively breaks the endothelial barrier of the tumor vasculature in HT1080 fibrosarcoma and A431 epidermoid carcinoma tumor models

**DOI:** 10.18632/oncotarget.25032

**Published:** 2018-04-27

**Authors:** Stephan Niland, Dorde Komljenovic, Jadranka Macas, Thilo Bracht, Tobias Bäuerle, Stefan Liebner, Johannes A. Eble

**Affiliations:** ^1^ Institute of Physiological Chemistry and Pathobiochemistry, Münster University Hospital, Münster, Germany; ^2^ German Cancer Research Center, Division Medical Physics in Radiology, Heidelberg, Germany; ^3^ Institute of Neurology, Edinger Institute, Johann Wolfgang Goethe University, Frankfurt, Germany; ^4^ Current address: Clinical Proteome Center, Ruhr-University, Bochum, Germany

**Keywords:** endothelial barrier, abnormal tumor vasculature, vasculogenic mimicry, rhodocetin-αβ, neuropilin-1

## Abstract

The tumor vasculature differs from normal blood vessels in morphology, composition and stability. Here, we describe a novel tumor vessel-disrupting mechanism. In an HT1080/mouse xenograft tumor model rhodocetin-αβ was highly effective in disrupting the tumor endothelial barrier. Mechanistically, rhodocetin-αβ triggered MET signaling via neuropilin-1. As both neuropilin-1 and MET were only lumen-exposed in a subset of abnormal tumor vessels, but not in normal vessels, the prime target of rhodocetin-αβ were these abnormal tumor vessels. Consequently, cells lining such tumor vessels became increasingly motile which compromised the vessel wall tightness. After this initial leakage, rhodocetin-αβ could leave the bloodstream and reach the as yet inaccessible neuropilin-1 on the basolateral side of endothelial cells and thus disrupt nearby vessels. Due to the specific neuropilin-1/MET co-distribution on cells lining such abnormal tumor vessels in contrast to normal endothelial cells, rhodocetin-αβ formed the necessary trimeric signaling complex of rhodocetin-αβ-MET-neuropilin-1 only in these abnormal tumor vessels. This selective attack of tumor vessels, sparing endothelial cell-lined vessels of normal tissues, suggests that the neuropilin-1-MET signaling axis may be a promising drugable target for anti-tumor therapy, and that rhodocetin-αβ may serve as a lead structure to develop novel anti-tumor drugs that target such vessels.

## INTRODUCTION

The tumor vasculature which supports tumor metabolism, growth, and hematogenic metastasis differs from vessels found in normal tissues. It appears as a disorganized tangle of shabby blood vessels with various structural abnormalities, such as heterogeneous diameter and shape, bulges, dead ends, arterio-venous shunts, plasma channels lacking blood cells, and it may even have a discontinuous endothelial cell lining [1, 2]. Abnormal tumor blood vessels (ATV) can be classified into at least six distinct types [1], and even tumor cell-lined blood cell-filled conduits have been described in some tumors [3]. Such vasculogenic mimicry (VM) is not found in the healthy body but is unique to tumor tissue where it promotes cancer growth and hematogenic dissemination of detaching tumor cells causing metastasis [4, 5]. Thus, it is associated with poor prognosis [6, 7]. Instead of angiogenic ECs, in VM highly invasive and genetically dysregulated tumor cells mimic ECs and partially or fully line vascular tubes to form fluid-conducting channels that supply the tumor with blood [8]. The concept of VM has been initially viewed critically [9]. Meanwhile VM has been observed in many cancers, such as astrocytoma WHO grade II-III [10] glioblastoma (astrocytoma WHO grade IV) [11], melanoma [12], cancers of breast [13], gallbladder [14] pancreas [15], liver [16], gastrointestinal [17] and colorectal tract [18], lung [7], ovaries [19], prostate [20], and various sarcomas [21, 22].

ATV and VM vessels have been suggested to be promising targets for drug delivery and antitumor therapy [1, 2, 23], in particular as the lack of ECs in VM-featuring tumors may be partially responsible for their resistance to treatment with anti-angiogenic agents, e.g. angiostatin or endostatin [24], or to VEGF inhibition [25].

Rhodocetin, a heterotetrameric C-type lectin-like protein of *Calloselasma rhodostoma* venom, is an inhibitor of α2β1 integrin [26, 27]. To test the effect of rhodocetin on growth and constitution of integrin α2β1-expressing solid tumors *in vivo*, we chose a murine tumor xenograft model, in which human HT1080 cells form aggressive angiogenic tumors and abundantly present collagen-binding integrin α2β1 on their surface. Moreover, VM by HT1080 has been demonstrated by expression of green fluorescent protein in them [28]. Rhodocetin completely inhibits HT1080 adhesion to collagen-I in the desmoplastic tumor environment [27]. Moreover, rhodocetin is a full antagonist of α2β1 integrins, as it turns off α2β1 integrin signaling and thus impedes stromal tumor invasion [29]. By blocking α2β1 integrin-mediated cell-matrix interactions, it also reduces metastasis [30]. However, only the γδ-subunit of the heterotetrameric rhodocetin was identified as α2β1 integrin inhibitor [31]. Its αβ-subunit interacts with neuropilin-1 (NRP1) on ECs and does not interfere with α2β1 integrin [32].

By binding to NRP1 rhodocetin-αβ stimulates EC motility *in vitro* by triggering the association of NRP1 with MET, thus promoting paxillin Y31 phosphorylation [32]. The interconversion of paxillin causes the transformation of focal adhesions into much smaller focal complexes. This, together with a restructuring of the actin cytoskeleton, stimulates cell motility independent from α2β1 [32]. Thus, rhodocetin-αβ-mediated NRP1-MET signaling increases endothelial cell (EC) motility [32]. Moreover, in HT1080 cells *NRP1* is upregulated under hypoxia, along with other angiogenic markers in a mouse xenograft tumor model, in which HT1080 cells form functional vasculogenic mimicry vessels [28].

Here we investigated the vessel-disrupting effect of rhodocetin-αβ on the tumor endothelial barrier in an HT1080 fibrosarcoma xenograft mouse model and confirmed this effect using an A431 epidermoid carcinoma xenograft mouse model.

## RESULTS

### Rhodocetin αβ induces tumor hemorrhage

**Figure 1:**
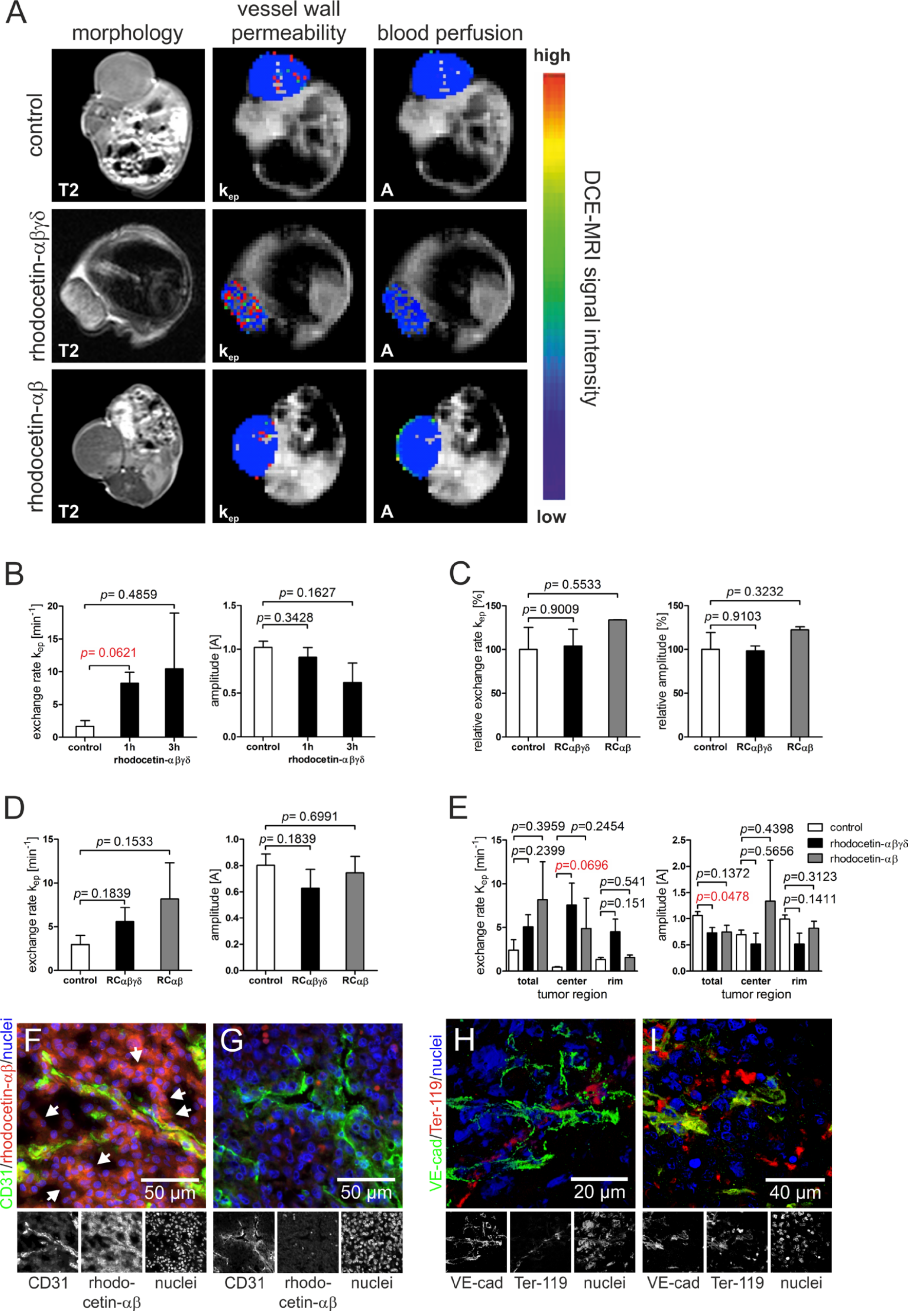
Rhodocetin-αβ induces hemorrhage in tumor tissue (**A**) vessel perfusion and permeability of xenograft tumor mice was monitored by DCI-MRI three hours after treatment with rhodocetin- αβγδ and rhodocetin-αβ. The effect on HT1080 tumors is shown in T2-weighted and DCE-MRI scans. (**B**) DCE-MRI parameters, exchange rate k_ep_ (vessel wall permeability) and amplitude A (blood perfusion, relative blood volume), before (control), and 1h and 3h after injection of rhodocetin-αβγδ showed no change in blood perfusion but a time-dependent increase in vessel wall permeability. (**C**) vessel permeability and blood flow in muscle vasculature did not change after injection of rhodocetin tetramer or its αβ-subunit. K_ep_ and A values relative to control values and SEM are shown. Control: injection of PBS. (**D**) in tumor tissue, tetrameric rhodocetin and its αβ-subunit caused a strong increase in k_ep_, whereas the relative blood volume did not change. Control: injection of PBS. (**E**) tetrameric rhodocetin and also its αβ-subunit enhanced vessel permeability (k_ep_) especially in the tumor center as compared to its periphery (rim), whereas only rhodocetin-αβ slightly increased blood flow (A) in the tumor center. Control: injection of PBS. Data represent mean with SEM. (**F**) rhodocetin-αβ was detectable in tumors in reticular structures (arrows) outside of blood vessels (CD31, green) with biotinylated mAb VIIF9 (red). (**G**) these structures were not labeled when the rhodocetin-αβ-specific primary antibody was omitted. CD31 on ECs is labeled green, rhodocetin-αβ red, and nuclei blue. The detection of rhodocetin-αβ on endothelial and tumor cells demonstrates the presence of a rhodocetin-αβ receptor. (**H**) blood cells within a VE-cadherin^−^ conduit in the tumor center were detected with an antibody against the lineage marker Ter-119 (red). (**I**) rhodocetin-αβ treatment results in massive extravasation of blood cells in the tumor center. VE cadherin green, Ter-119 red, nuclei blue. Original magnification was 400× (**F**–**G**) and 630× (**H**–**I**). Representative images are shown.

To test the effect of rhodocetin on solid tumors of HT1080 fibrosarcoma cells, rhodocetin was injected in tail veins of tumor-bearing mice. Solid tumors became hemorrhagic within 1–3 hours (Supplementary Figure 1), while no obvious hemorrhage was detectable in other tissues, such as skin, muscle, kidney, or liver. This was likewise observed in an A431 epidermoid cell xenograft mouse tumor model (data not shown). Remarkably, we observed that the NRP1-binding rhodocetin-αβ without the α2β1 integrin-blocking rhodocetin-γδ-subunit, was sufficient for this effect. For intra-vital measurement, dynamic contrast-enhanced magnetic resonance imaging (DCE-MRI) was employed. Three hours after tail-vein injection, DCE-MRI of tumor-bearing mice revealed that intravenously administered rhodocetin and also its αβ-subunit on its own (i) selectively accumulated in the tumor tissue, (ii) reduced its blood perfusion, and at the same time (iii) increased the vessel permeability/leakage of tumor vessels, while (iv) vessels of other tissues, such as muscle (Figure 1C), were unimpaired (Figure 1A–1D). Vessel perfusion (amplitude A, relative blood volume) and vessel wall permeability (exchange rate k_ep_) were overlaid on T2-weighted morphologic images. Rhodocetin-αβ-induced vessel leakage was especially pronounced in the hypoxic core where also the blood volume was increased by rhodocetin-αβ (Figure 1E), whereas k_ep_ in control tissue (muscle) was unaffected (Figure 1C).

### Intravenously injected rhodocetin-αβ is detectable in reticular structures within tumor tissue

To investigate the role of rhodocetin-αβ in tumor-specific vessel disruption we analyzed the presence of rhodocetin-αβ within tumor tissue three hours after intravenous application. In tumor tissue, rhodocetin-αβ was not only present within CD31-positive blood vessels, but it was also found in and around abundant CD31-negative reticular structures (Figure 1F, 1G). Also in A431 cells, rhodocetin-αβ was detectable in CD31-negative reticular patterns (Supplementary Figure 3A, 3B). This observation of rhodocetin-αβ-containing reticular structures indicated a conduit system other than EC-lined vessels in both tumor types. Moreover, immunohistochemistry of HT1080 and A431 tumors demonstrated the presence of rhodocetin-αβ receptors on both tumor and endothelial cells. Interestingly, endothelial cells were labeled with rhodocetin not on their apical surface but rather at their basolateral side (Supplementary Figure 2A’, 2B), providing evidence for a differential subcellular distribution of a rhodocetin-αβ receptor to different membrane compartments.

### Non-EC-lined vascular structures in tumor tissue contain blood cells

To assess the functional involvement of such non-EC-lined reticular structures in blood transport, blood cells were visualized with an erythroid lineage marker. In contrast to other tissues, such as skin, muscle, kidney and liver, blood cells in tumor tissue were not only found within normal blood vessels as identified by VE-cadherin, but they were detectable also in VE-cadherin-negative conduits in HT1080 and A431 tumors (Figure 1H, Supplementary Figure 3C). Hence, these abnormal vascular-like structures suggest vasculogenic mimicry. Remarkably, rhodocetin-αβ caused the total disappearance of such conduits and a spill of red blood cells throughout the tumor tissue (Figure 1I, Supplementary Figure 3D).

### Rhodocetin-αβ opens abnormal tumor vessel walls

**Figure 2:**
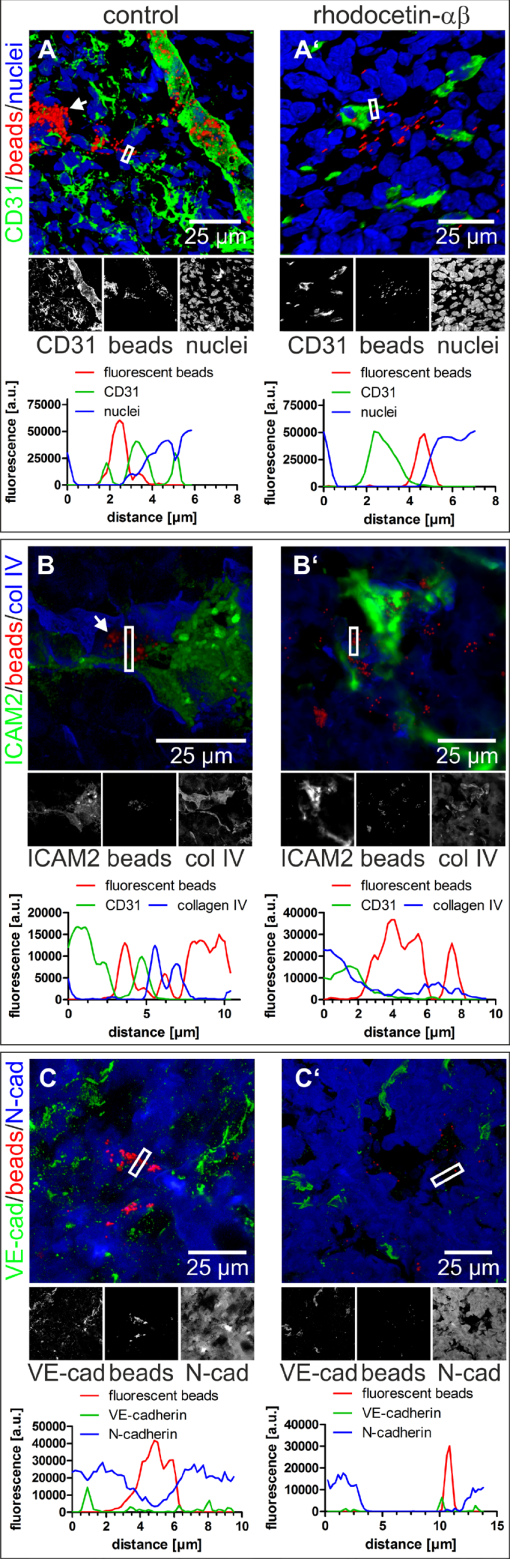
Nano-beads leak from tumor blood vessels after treatment with rhodocetin-αβ (**A**–**C**) intravenously injected 100 nm-beads were confined to regular blood vessels and also to hollow structures that are not lined by ECs (white arrow). **A’**, **B’**, **C’** treatment with rhodocetin-αβ leads to leakage of beads. CD31 (**A**, **A’**), ICAM2 (**B**, **B’**), and VE-cadherin (**C**, **C’**) highlight ECs in green. Collagen IV delineates basement membranes in blue (**B**, **B’**) and N-cadherin antibodies detect HT1080 cells (**C**, **C’**) labeled blue. Nano-beads are shown in red throughout. Nuclei are stained blue in **A**, **A’**. In **A**, **A’**, **B**, and **B’**, EC-lined tumor blood vessels (marked by white-bordered rectangles) were line-scanned for their fluorescence intensity. Note, that beads in rhodocetin-αβ-free controls, but not in rhodocetin-αβ-treated samples, were confined between flanking EC markers in green (CD31 in **A**, and ICAM2 in **B**). In **C**, **C’**, tumor vessels lacking EC markers, but consisting of N-Cadherin-expressing HT1080 were examined. Line scans of fluorescence intensity VM vessels (indicated by a white rectangle) demonstrated that beads were completely enclosed in VM vessels in PBS-treated controls (**C**), but were almost completely outside of N-Cadherin-confined areas after rhodocetin-αβ treatment (**C’**). Original magnification was 400× (**A**) and 630× (**A’**, **B-B’**, **C-C’**). Representative images are shown.

To determine the extent of rhodocetin-αβ-induced tumor vessel leakage, FluoSpheres were intravenously injected into tumor-bearing mice two minutes before the animals were sacrificed. The FluoSpheres were predominantly distributed throughout the CD31^+^, ICAM2^+^, and VE-cadherin^+^ vasculature (Figure 2A–2C). Moreover, these nano-beads were found in partly or completely CD31^–^, ICAM2^-^, and VE-cadherin^−^ vessel-like structures (Figure 2A, 2B, arrows). Instead, these vessel-like structures were positive for N-cadherin, which is expressed by HT1080 cells [33–35] (Figure 2C). After treatment with rhodocetin-αβ, numerous nano-beads were outside of CD31^+^, ICAM2^+^, and VE-cadherin^+^ EC-lined vessels. Moreover, N-cadherin^+^ conduits were disrupted and failed to retain FluoSpheres (Figure 2A’–2C’).

**Figure 3:**
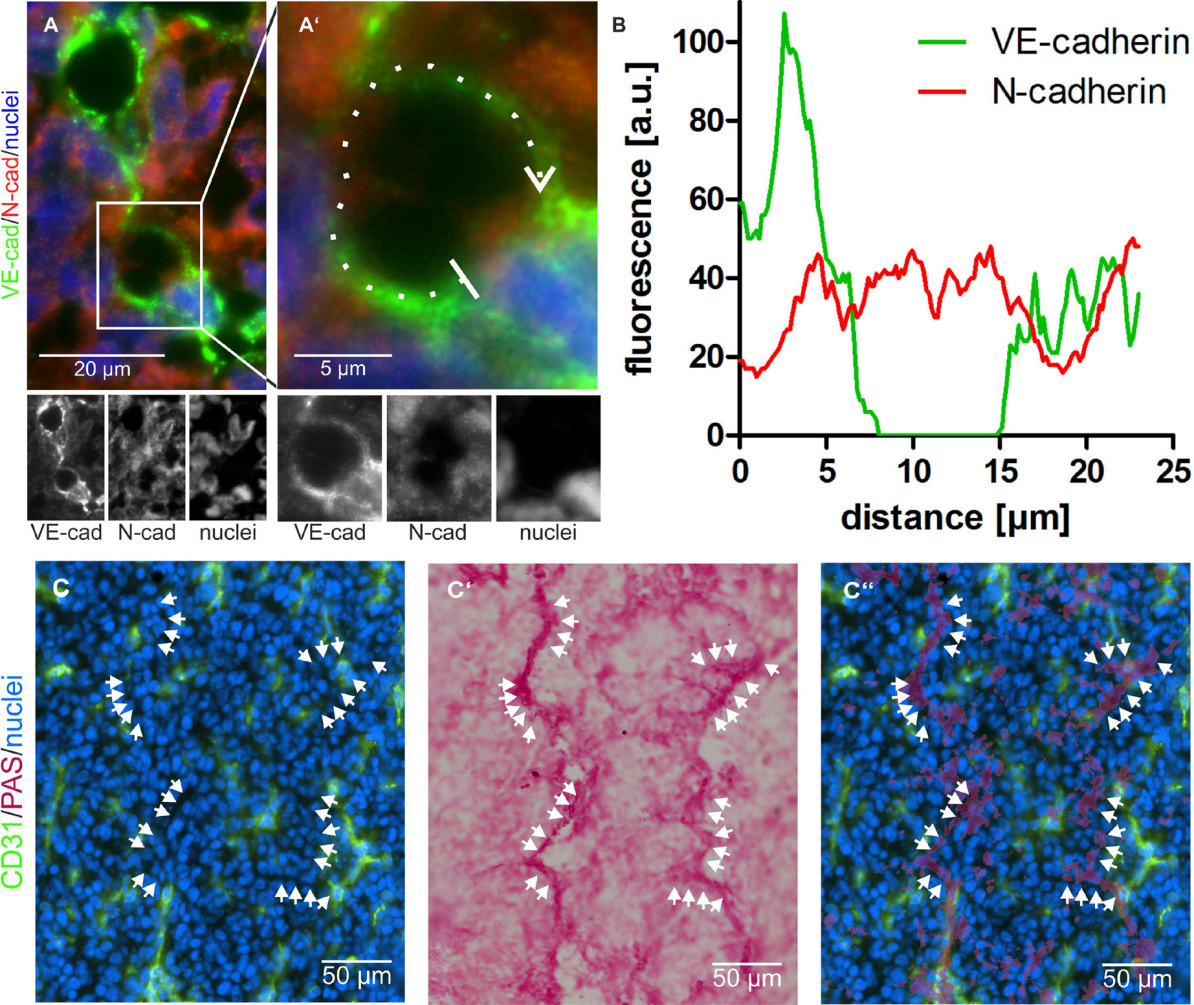
Vasculogenic mimicry in tumor tissue (**A**) Abnormal tumor vessels are lined at least in part by VE-cadherin negative and N-cadherin positive tumor cells. Cropped at higher magnification in A’, VE-cadherin is labeled in green and N-cadherin in red. (**B**) fluorescence intensity of both signals along the dotted line marked in **A’** in clockwise direction and averaged over a width of five pixels shows the absence of the VE-cadherin signal at the left vessel wall. **C**–**C’’**, CD31-negative/PAS-positive VM vessels were visualized in tumor tissue by consecutive immunostaining and histochemical staining of the same cryosection: Normal CD31-positive blood vessels are labeled in green (**C**, **C’’**), whereas CD31-negative VM vessels are detectable by PAS staining (**C’**, **C’’**). Nuclei are stained blue. Cryosections were first immunostained and photographed (**C**), subsequently histochemically PAS-stained and photographed again (**C’**), and then the images were overlaid to demonstrate numerous CD31-negative/PAS-positive VM vessels (**C’’**, arrows). Original magnification was 400× (**A**–**A’**) and 200× (**C**–**C’’**). Representative images are shown.

In tumor sections, vessel-like structures were detected, in which VE-cadherin was virtually absent from N-cadherin^+^ areas showing that N-cadherin^+^/VE-cadherin^−^ HT1080 cells can integrate into a VE-cadherin^+^ EC layer (Figure 3A, 3B). The same could be observed in A431 tumors (Supplementary Figure 4A, 4A’, and 4B). In both cases tumor and endothelial cells were in direct contact to blood at sites of ATV/VM in tumor tissue. VM vessels are commonly characterized by their CD31^–^/periodic acid-Schiff^+^ (PAS^+^) staining. Numerous CD31^–^/PAS^+^ structures were detectable in HT1080 tumors (Figure 3C–3C’’, arrows) as well as in A431 tumors (Supplementary Figure 4C–4C’’, arrows).

### Rhodocetin-αβ-induced vessel damage is tumor-selective

**Figure 4:**
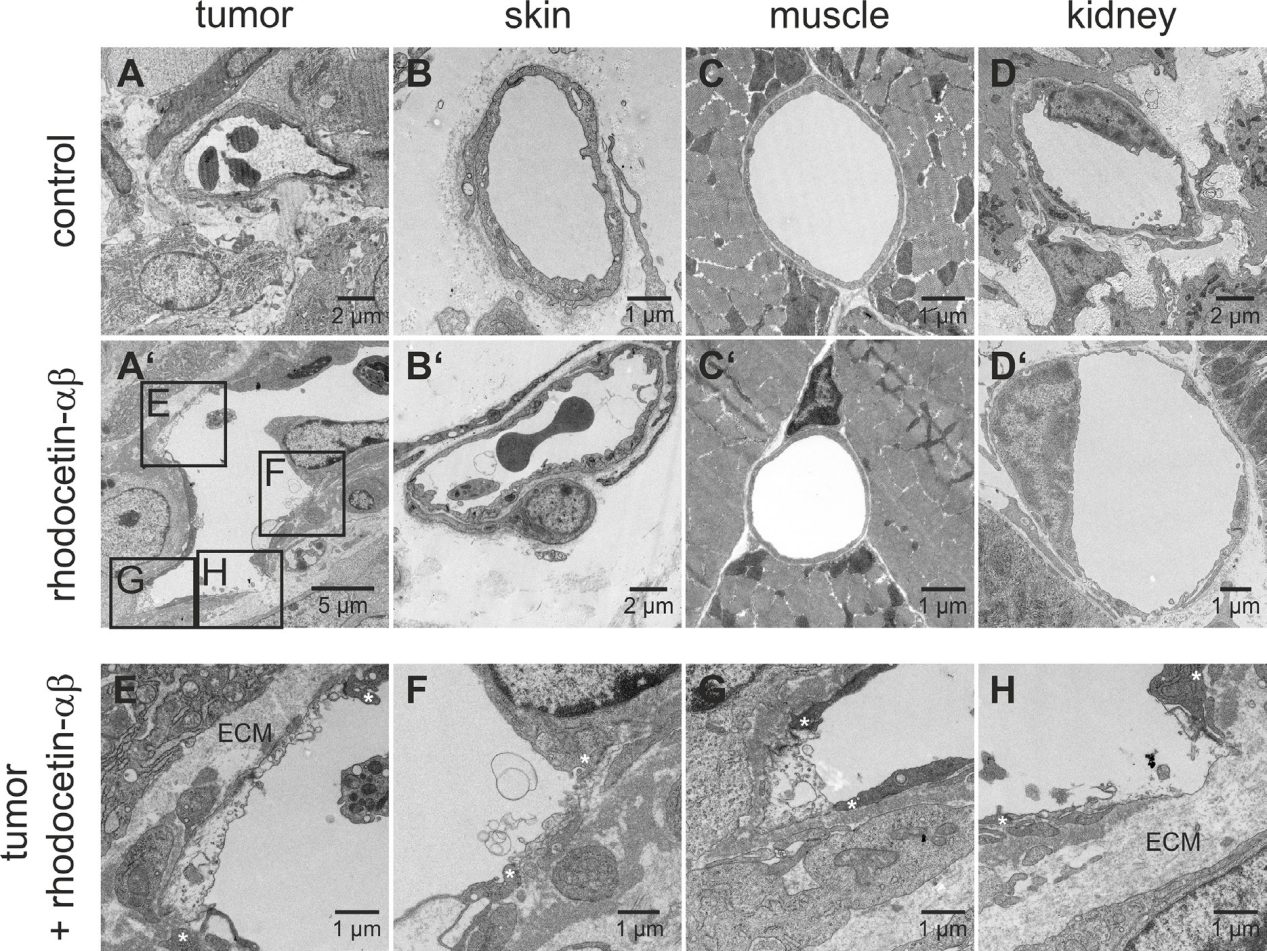
Rhodocetin-αβ-induced vessel damage is tumor-selective Transmission electron micrographs show blood vessels of PBS-treated tumor (**A**) and the demise of tumor ECs three hours after intravenous injection of rhodocetin-αβ (**A’**). Endothelia in other tissues (**B**, skin, **C**, muscle, and **D**, kidney) were unaffected by rhodocetin-αβ (**B’**, **C’**, and **D’**). In tumor, rhodocetin-αβ caused EC detachment and lead to denuded basement membrane. (**E**–**H**) magnified regions from affected areas in **A’** showing the damage of ECs after treatment with rhodocetin-αβ. Asterisks mark the borders of endothelial lesions, which even lead to denuded basement membrane (**H**); ECM, extracellular matrix. Original magnification was 1900× (**A’**); 2900× (**A**, **B’**, **D**), 4800× (**C’**, **D’**, **F**), and 6800× (**B**, **E**, **G**, **H**). Representative images are shown.

To analyze the tumor-specificity of rhodocetin-αβ, tissue samples were subjected to ultrastructural analysis. After treatment with rhodocetin-αβ, ECs in skin (Figure 4B, 4B’), muscle (Figure 4C, 4C’), and kidney (Figure 4D, 4D’) remained attached to their basement membrane and kept up a tight vessel seal, whereas in tumor tissue massive damage of ECs occurred (Figure 4E–4G), resulting in areas of denuded basement membrane (Figure 4H). Such lesions would allow leakage of serum and blood-borne rhodocetin-αβ, explaining the reticular staining in Figure 1F and Supplementary Figure 3D. Even nano-bead-sized particles (Figure 2) and eventually blood cells (Figure 1I, Supplementary Figure 3D) may leave such damaged vessels.

### NRP1, the target of rhodocetin-αβ, is accessible from the vessel lumen only on VM tumor cells

**Figure 5:**
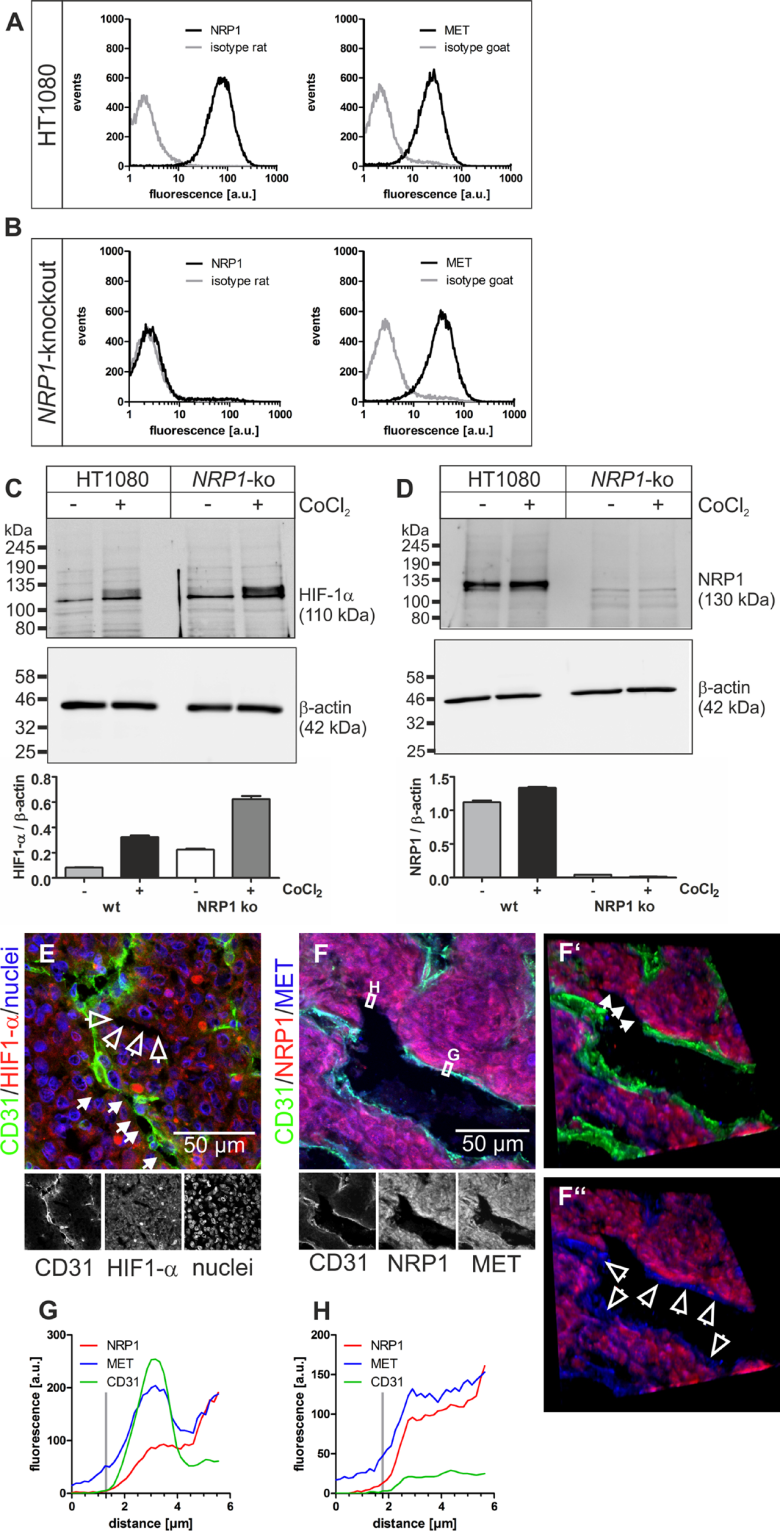
NRP1 and MET on HT1080 cells are accessible for blood-borne rhodocetin-αβ (**A**) expression of *NRP1* and *MET* by HT1080 cells was proven by flow cytometry. Gray, isotype-matched controls. (**B**) flow cytometry of *NRP1*-knockout HT1080 cells demonstrating their NRP1-deficiency and unaffected *MET* expression. (**C**) treatment with CoCl_2_, mimicking a hypoxic tumor micro-environment, induced upregulation of HIF-1α. (**D**) *NRP1*, as a downstream target of HIF-1α, is upregulated in HT1080 cells but not in *NRP1*-knockout HT1080 cells. β-actin immunoblots show even loading. (**E**) increased HIF-1α (red) levels in hypoxic tumor regions, which also contained partly (arrows) or completely (open arrows) EC-deficient VM vessels. ECs are stained green and nuclei blue. (**F**) immunostaining of NRP1 (red) and MET (blue) showed that both proteins were present on HT1080 cells and ECs in tumor tissue. Note the continuity between EC-lined vasculature and EC marker-deficient vessels (arrows in F’). **F’**, **F’’**, in oblique view, gating of the green CD31 signal also showed an apical absence of NRP1 on ECs in contrast to MET (open arrows). (**G**) the fluorescence intensity along a traceroute, averaged over a width of 5 pixels, (rectangle in **F**) through the endothelium revealed that in ECs NRP1, unlike MET, is absent from the apical side and restricted to the basolateral side. In contrast, on ATV/VM-lining cells (**F’**, arrows) both NRP1 and MET are accessible from the bloodstream (**H**). Vertical gray lines in **G** and **H** indicate the position of the apical cell border. Original magnification was 400× (**E**) and 630× (**F-F’’**). Representative images are shown.

**Figure 6:**
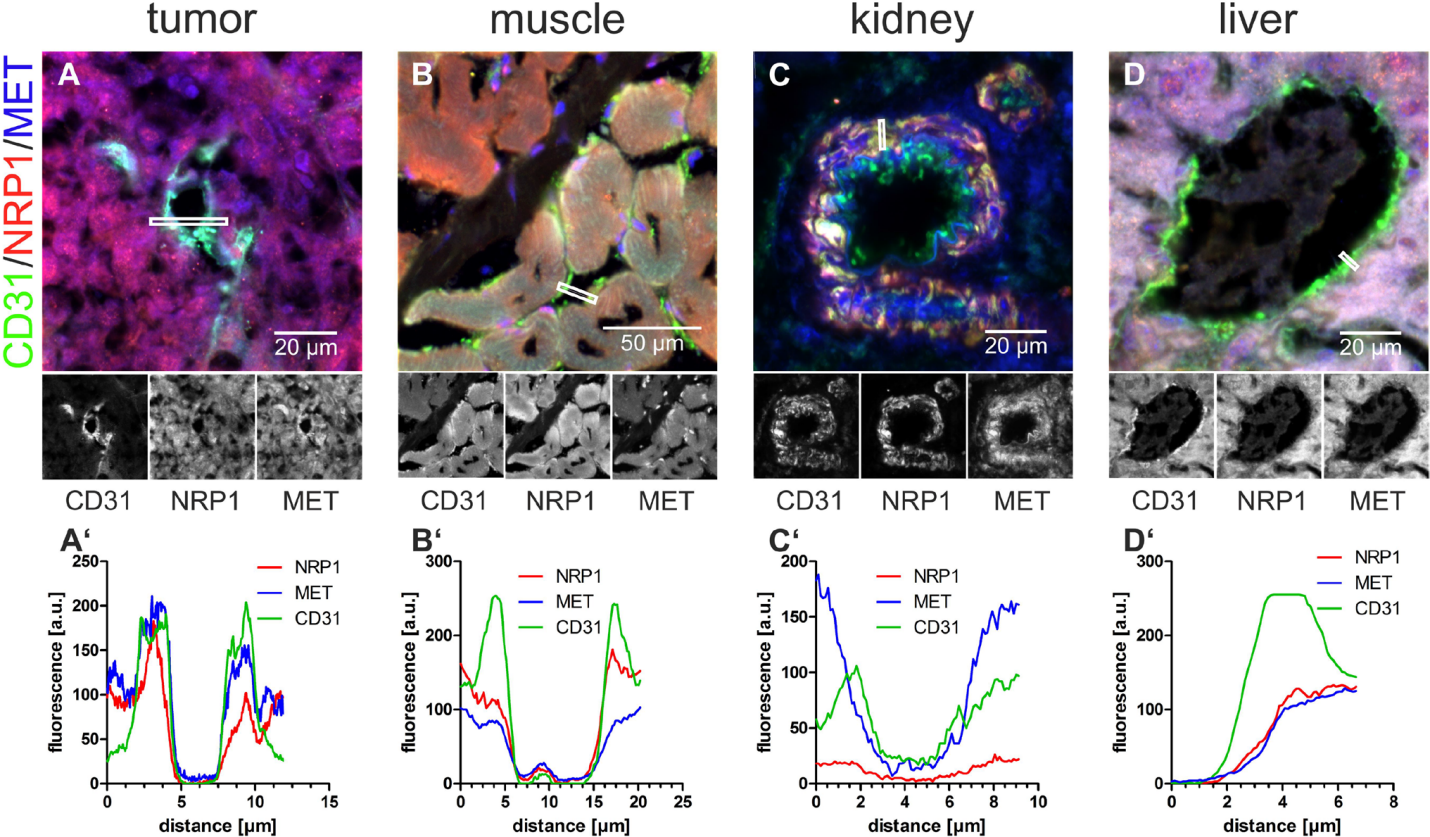
In normal blood vessels, blood-borne rhodocetin-αβ cannot elicit the interaction of NRP1 with MET (**A**–**D**), the EC-marker CD31 (green), NRP1 (red), and MET (blue) in cryosections of tumor (A), muscle (B), kidney (C), and liver (D) were analyzed for their subcellular distribution. **A’**, fluorescence intensities, averaged over a width of 5 pixels, along the lines marked by white rectangles showed that NRP1 (red line) is, in contrast to MET (blue line), only present on the basolateral side of ECs. **B’**, in muscle, NRP1 but not MET is present on the apical face of ECs. **C’**, in kidney, MET is present but NRP1 is missing on the luminal face of ECs. **D’**, in liver, both NRP1 and MET are absent from the apical face and merely accessible via the space of Disse. Original magnification was 630× (A, C, D) and 400× (B). Representative images are shown.

To understand why rhodocetin-αβ attacks selectively the tumor vasculature, we investigated its biochemical mode of action. In ECs, rhodocetin-αβ induces cell motility via NRP1/MET-signaling [32]. In addition, HT1080 fibrosarcoma and A431 epidermoid carcinoma cells likewise expressed both NRP1 and MET (Figures 5A, 5F, 6A; Supplementary Figure 7B) especially under chemically induced hypoxia (Figure 5C, 5D, Supplementary Figure 6), thus being potential targets of rhodocetin-αβ as well.

HIF-1α-staining verified hypoxia in central HT1080 and A431 tumor areas (Figure 5E; Supplementary Figure 7A), where vessel wall damage by rhodocetin-αβ was especially prominent (Figure 1E), and abundant composite/ATV/VM vessels were just partially EC-lined (Figure 5E and Supplementary Figure 7A, arrows) or even completely devoid of ECs (Figure 5E and Supplementary Figure 7A, open arrows).

In tumors, NRP1 was restricted to the basolateral side of ECs (Figures 5F, 5G and 6A, 6A’, red line, Supplementary Figure 7B, 7C) and was virtually absent from their apical side (Figure 5F, 5F’, 5F’’), whereas MET was present on both faces of ECs (Figures 5F, 5G and 6A, 6A’, blue line, Supplementary Figure 7B, 7C). Therefore, NRP1 on vascular ECs was not accessible to blood-borne rhodocetin-αβ, ruling out that it targets ECs of tumor vessels primarily. This reflected the observation that unlike in HT1080 and A431 tumor cells (Figure 1F, Supplementary Figure 3A) rhodocetin-αβ bound only to the basolateral side of ECs (Supplementary Figure 2A’, 2B). In contrast, HT1080 cells at sites of VM or in ATV which are negative for common EC markers did not show any differential compartmentalization of NRP1 and MET on their surface (Figure 5F, 5H, Supplementary Figure 7B, 7D). In a rotated view the EC-free lining of a VM vessel is evident (Figure 5F’, arrows) and gating of the CD31 signal revealed the presence of MET but not of NRP1 on the apical side of ECs in contrast to VM vessel-lining HT1080 cells (Figure 5F’, 5F’’, open arrows). Figure 5F also provides strong evidence that VM and normal blood vessels actually do anastomose.

Comparative immunohistochemistry of tumor and other tissues revealed why rhodocetin-αβ did not exert any effect in other organs. Vascular ECs in muscles presented NRP1 on their apical side, but its interaction partner MET was confined to their basolateral side (Figure 6B, 6B’), ruling out a functional rhodocetin-αβ-NRP1-MET signaling complex on the luminal face of ECs in muscle tissue. Conversely, on kidney vascular ECs, MET but not NRP1 as the receptor for rhodocetin-αβ was detected (Figure 6C, 6C’). In contrast, hepatocytes express both NRP1 and MET towards the perisinusoidal space (Figure 6D, 6D’). Hence, both are accessible to blood-borne rhodocetin-αβ via liver sinusoids with their fenestrated, discontinuous endothelium (Supplementary Figure 5). The possible formation of a functional rhodocetin-αβ-NRP1-MET signaling complex on hepatocytes correlates with an occasional electron microscopic observation of hepatic EC damage (Supplementary Figure 5). Only in the cell lining of tumor VM vessels both NRP1 and MET were directly accessible from the vessel lumen (Figure 6A, 6A’).

### Rhodocetin-αβ increases motility of VM tumor cells via NRP1

**Figure 7:**
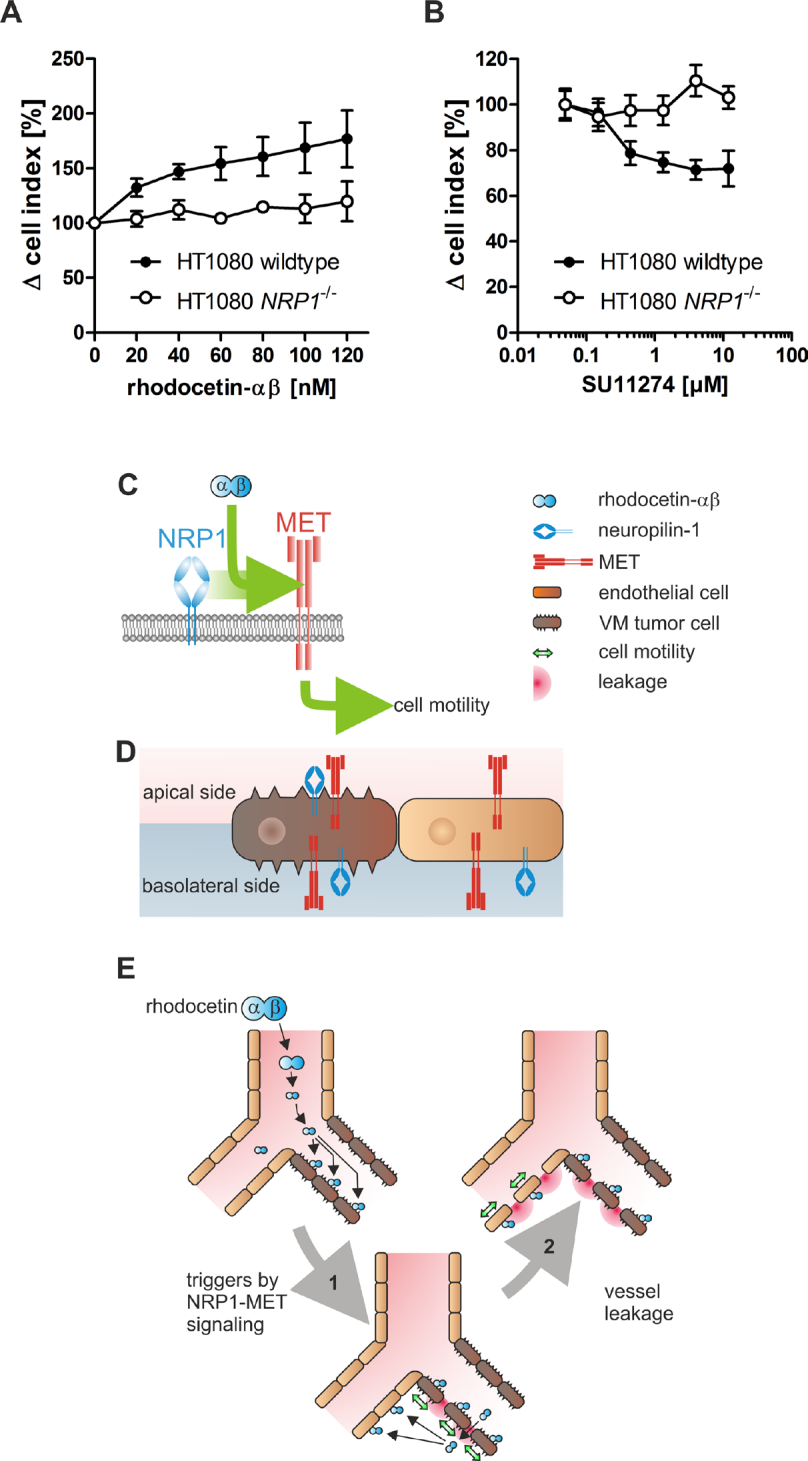
HT1080 cells are susceptible to rhodocetin-αβ (**A**) only *NRP1* expressing HT1080 cells respond to rhodocetin-αβ. The dose-dependent effect of rhodocetin-αβ on migration of *NRP1* expressing (filled symbols) and NRP1-deficient HT1080 cells (open symbols) was monitored with an xCELLigence system. Migration rate towards a rhodocetin-αβ source was expressed as Δ cell index normalized to unstimulated control. Data represent mean ± SEM of four experiments with duplicates. (**B**) rhodocetin-αβ-induced migration of HT1080 cells depends on the activity of MET. Migration towards 60 nmol/L rhodocetin-αβ was challenged with the MET inhibitor SU11274. In *NRP1* expressing HT1080 cells, SU11274 dose-dependently inhibited rhodocetin-αβ-elicited cell migration in contrast to NRP1-deficient cells. (**C**, **D**, **E**) scheme, depicting the mechanism of rhodocetin-αβ-induced tumor hemorrhage. (C) rhodocetin-αβ recruits NRP1 to MET, which triggers via paxillin-Y31 phosphorylation adhesome restructuring from focal adhesions to focal complexes, thus initiating a motile phenotype. (D) in this way blood-borne rhodocetin-αβ induces a motile phenotype in VM vessel-lining tumor cells. Only on the tumor cells the subcellular distribution of NRP1 and MET allows formation of a ternary complex with blood-borne rhodocetin-αβ. (E) thereby, the walls of already weak VM vessels become more permeable (1). Rhodocetin-αβ reaches the basolateral side of neighboring ECs and likewise induces their motility (2). Consequently, at sites of VM also normal vessels become increasingly leaky, eventually resulting in breakdown of the vessel wall barrier and massive hemorrhage solely in tumor tissue.

To test whether HT1080 cells lining VM vessels are susceptible to rhodocetin-αβ, we studied its promigratory effect on HT1080 cells (Figure 7A, 7B). Rhodocetin-αβ dose-dependently stimulated migration of NRP1^+^ but not of NRP1-deficient (Figure 5B–5D) HT1080 cells (Figure 7A). Treatment with the MET inhibitor SU11274 reduced rhodocetin-αβ-induced migration of NRP1^+^ but not of NRP1-deficient HT1080 cells, even under chemically induced hypoxic conditions (Figure 7B).

## DISCUSSION

Blood-borne rhodocetin-αβ triggers NRP1-MET signaling in tumor cells lining ATV/VM vessels, which consequently become increasingly permeable. Thus, after leaking into the tissue and approaching nearby ECs from their basolateral side, rhodocetin-αβ can elicit the same signaling cascade in these ECs [32]. This causes the breakdown of the vessel wall barrier in tumor tissue without harming the vasculature in other tissues. Our data strongly point towards a disruption of VM vessels by rhodocetin-αβ. Yet, tumor endothelial cells (TECs) are heterogeneous and can originate from multiple sources [36]. Although VM by HT1080 has been demonstrated by expressing green fluorescent protein in them [28], it cannot be ruled out that any abnormal TECs lacking accepted EC markers, e.g. after endothelial-mesenchymal transition, and showing a similar subcellular distribution of NRP1 and MET, may build up ATVs, which would likewise be targeted by rhodocetin-αβ in a tumor-selective manner. Comprehensively, in the discussion of our findings we combine this conceivable scenario with VM.

Being not cytotoxic and having no adverse side effects in previous studies [29, 30] rhodocetin, particularly its αβ-subunit, caused pronounced hemorrhage in tumor-bearing mice without any noticeable effect on healthy mice. Strikingly, hemorrhage was restricted to tumor tissue, although in liver, rhodocetin-αβ caused electron-microscopically detectable cell damage. At sites of tumor vessel leakage, rhodocetin-αβ was found within and notably outside of blood vessels, where it sometimes occurred in reticular structures, which could not be labeled with antibodies against EC markers. This renders their assembly by abnormal TECs unlikely, and suggests that these are VM structures. Rhodocetin-αβ may breach the diffusion barrier of such tubes by inducing motility in the lining tumor cells via NRP1-MET signaling, as it was previously described for ECs *in vitro* [32]. Moreover, the intrinsic leakiness of ATVs [1] is conspiring. Binding of rhodocetin-αβ to HT1080 and to A431 cells demonstrated the presence of a cognate receptor. Moreover, blood cells in VE-cadherin-negative vascular-like structures provided strong evidence that in tumor tissue VM in addition to ATVs supports the blood supply. Absence of VE-cadherin in VM/ATVs foils their tight vessel seal, because VE-cadherin is characteristic of endothelial cell-cell contacts, whereas N-cadherin is responsible for the anchorage to surrounding cells [37, 38].

Leakiness of ATVs is strongly enhanced by rhodocetin-αβ. Platelets prevent intratumor hemorrhage and their depletion leads to rapid destabilization of tumor vessels [39]. Although glycoprotein Iα (GPIbα) is a receptor for rhodocetin-αβ on platelets [40], sole inhibition of GPIbα on platelets does not cause intratumor hemorrhage [39]. Thus, the observed rhodocetin-αβ-increased vessel leakiness was caused by another effect, although a synergistic effect of GPIbα inhibition on platelets may be involved.

To characterize this rhodocetin-αβ-induced tumor vessel wall permeability more thoroughly, nano-beads were used. ATVs including VM vessels have variable calibers, sometimes even they are too narrow for erythrocytes to squeeze through, yet capable to transport plasma and contribute to oxygenation of tumor tissue by extraerythrocytic hemoglobin from ruptured erythrocytes [8, 41, 42].

Without rhodocetin-αβ, intravenously injected nano-beads were predominantly located within CD31 and ICAM2 expressing normal blood vessels. In addition, they could also be observed in high density in cross- and longitudinally-cut tube-like structures, which were negative for endothelial markers, but showed the mesenchymal marker N-cadherin, which is expressed in HT1080 cells [35]. This implies a lining of these conduits by HT080 cells. After application of rhodocetin-αβ, extravascular beads were scattered over the entire tumor section, and neither cross- nor longitudinally-cut tube-like structures containing nano-beads were detectable. HT1080, and likewise A431, evidently express both NRP1 and MET all over their plasma membrane. Hence, on the luminal surface of such conduits blood-borne rhodocetin-αβ can bind to NRP1 and thereby trigger MET signaling. The latter promotes cell motility, and the increased load on cell-cell contacts renders such conduits leaky. This suggests that these tube-like structures were the first to disintegrate. Subsequently, rhodocetin-αβ leaking from such conduits could reach the basolateral side of neighboring endothelial cells. Thus, endothelial cells with their exclusively basolateral NRP1 expression could also bind rhodocetin-αβ via NRP1 and thereby trigger MET signaling. As a consequence of this basolateral attack by rhodocetin-αβ, the endothelial barrier breaks down, too, eventually leading to massive hemorrhage.

In tumor tissue, rhodocetin-αβ destroyed the endothelial barrier by transforming integrin-containing adhesomes of ECs [32], thereby inducing EC detachment. Thus, patches of denuded basement membrane were opened, whereby more rhodocetin-αβ could leak from the vessel lumen and reach NRP1 on neighboring cells. In contrast, due to their better endothelial seal, EC-lined vessels in other regions of the body were unresponsive to rhodocetin-αβ apart from an off-target effect in liver sinusoids. After leaking from tumor blood vessels, rhodocetin-αβ might also interact with NRP1 on surrounding pericytes and thereby further destabilize the tumor vasculature. However, such a pericyte-based vessel destabilizing effect is unlikely, because on pericytes from human placenta (C-12980, PromoCell. Heidelberg, Germany) only NRP1 could be detected by flow-cytometry, but its essential interaction partner MET was not expressed on pericytes (data not shown).

Although an incorporation of abnormal TECs that do not express normal EC markers in leaky ATVs is conceivable, the enhanced *NRP1* expression in HT1080 cells under hypoxia, the apparent integration of HT1080 and of A431 tumor cells in the endothelial layer, and the concomitant subcellular localization of NRP1 and MET at the apical face of tumor cells are unique properties of VM. All three features explain the selective targeting of VM vessels by rhodocetin-αβ and illuminate the tumor vessel-selective activity of rhodocetin-αβ. These findings also suggest that VM vessels not only are the primary target of rhodocetin-αβ, but also serve as a gateway for blood-borne rhodocetin-αβ to the subendothelial space, where eventually it can access both NRP1 and MET of ECs. To this end, HT1080 and A431 cells must lose their contacts with neighboring tumor or endothelial cells by becoming motile. We demonstrated that VM vessels anastomose with normal blood vessels and thus are accessible to blood-borne rhodocetin-αβ. Such a continuity from normal to VM vessels adds another very important, if not the ‘smoking gun’ evidence for the existence of VM vessels [9].

In VM, tumor cells are surrounded by a basement membrane and thus form PAS-positive and CD31-negative tubular structures in tumor tissue [3, 6, 24]. Such CD31^–^/PAS^+^ VM vessels in addition to ATVs were abundant in tumor sections. Rhodocetin-αβ leaking from them, could reach NRP1 on the basolateral side of nearby ECs, and upon triggering EC motility destroy the barrier function of the vessel wall. In contrast, cells of other tissues were protected from rhodocetin-αβ by their subcellular compartmentalization of NRP1 and MET in different and not simultaneously accessible areas of their plasma membrane.

Migration of HT1080 cells along a rhodocetin-αβ gradient was concentration-dependent and could be inhibited by blocking MET, suggesting that rhodocetin-αβ induced tumor cell motility along the NRP1-MET signaling axis, similar as in ECs. Ablation of NRP1 on HT1080 cells canceled the promigratory effect of rhodocetin-αβ, and inhibition of MET did not affect migration of NRP1-deficient HT1080 cells. This implies the involvement of a rhodocetin-αβ-induced NRP1-MET signaling mechanism.

Rhodocetin-αβ can trigger NRP1-MET signaling in tumor cells, TECs, and endothelial cells alike. The tumor-specific incidence of VM/ATVs provides the basis for its selective destruction of the tumor vessel wall barrier. Only here both NRP1 and MET are in the same membrane compartment and exposed to the blood stream allowing their assembly in a ternary complex with blood-borne rhodocetin-αβ. This constellation is not found in vessels of normal tissues, such as skin, muscle, kidney, and liver. Based on these data we conclude that blood-borne rhodocetin-αβ triggers motility of tumor cells lining ATV/VM vessels (Figure 7C). Due to the subcellular localization of the corresponding receptors to the same membrane compartment only in tumor cells, this initiates tumor vessel disintegration (Figure 7D). As the walls of already weak ATV/VM vessels become more permeable, rhodocetin-αβ can reach the basolateral side of ECs within the tumor and likewise induce their motility (Figure 7E). Consequently, also EC-lined vessels in the vicinity of VM vessels become leaky with the final result of massive hemorrhage restricted to tumor tissue.

Rhodocetin-αβ-induced breakdown of the tumor vessel wall barrier may be relevant also for other *NRP1*- and *MET*-expressing malignancies in which VM occurs, for example glioma/astrocytoma [10, 43], prostate cancer [20, 44], and gastric cancer [17, 45]. Yet, in tumors of the gastrointestinal tract, an induction of hemorrhage represents a high risk of sepsis, and therefore such a vascular approach is contraindicated in this case. It is also not yet known whether an antivascular treatment strategy with a massive destruction of tumor vasculature may also cause dissemination of tumor cells. Obviously, there is the possibility that rhodocetin-αβ and drugs derived from its structure might promote metastasis. Hence, such a vascular disrupting approach is most likely feasible only in combination with other pharmaceuticals. While anti-angiogenic therapies to normalize the tumor vasculature [46] decrease the delivery of chemotherapeutics [47], a tumor-selective breakdown of the vessel wall barrier by tumor-vascular disrupting agents [2] may increase the efficacy of chemotherapeutics, which, due to an elevated interstitial pressure, reach their target area poorly. A high interstitial fluid pressure is substantially due to an increased permeability of ATVs, and the self-amplifying collapse of the tumor endothelial barrier by rhodocetin-αβ abrogates its semipermeability as a prerequisite for increased oncotic pressure.

Agents, such as rhodocetin-αβ, suggest that VM vessels may become a valid target in tumor therapy. Therefore, NRP1 may develop from a negative predictive factor to a positive selection marker for ATV/VM-targeted therapy. Systemic administration of anti-cancer drugs poses a major limitation to clinical efficacy. Induction of tumor-specific permeability of blood vessel walls via the NRP1-MET signaling axis may allow to use such agents at lower systemic dosage and therefore with better tolerance and less side effects.

## MATERIALS AND METHODS

### Materials and cells

Isolation of rhodocetin-αβ and corresponding antibodies were described previously [31]. The murine monoclonal antibody VIIF9 against rhodocetin-αβ was biotinylated with 0.25 mmol/L EZ-link sulfo-NHS-biotin (50-fold molar excess, Pierce/Thermo Scientific, Dreieich, Germany) in 1.5 ml PBS pH 7.4. After two hours at 21°C the reaction was stopped with 4 mmol/L Tris/HCl pH 7.6. Biotinylated VIIF9 was used at 2.5 μg/ml.

The following antibodies and chemicals were used at the specified dilutions: Rat anti-mouse CD31, clone MEC 13.3 (1:300, 550274, BD Biosciences, Heidelberg, Germany); rat anti-mouse ICAM-2, clone UZ10 (hybridoma supernatant, kind gift of R. Hallmann, Münster, Germany); rabbit polyclonal anti-mouse collagen IV (1:100, AB756P, Chemicon/EMD Millipore, Darmstadt, Germany); rabbit polyclonal anti-human N-Cadherin (1:50, 18203, Abcam, Cambridge, UK); goat anti-mouse VE-Cadherin (1:50, AF1002, R&D Systems, Wiesbaden, Germany); polyclonal goat anti-human NRP1 (C19) (1:300, SC7239, Santa Cruz, Heidelberg, Germany); polyclonal rat anti-human NRP1 (1:100, Pineda, Berlin, Germany), rabbit polyclonal anti-human MET (1:50, sc161, Santa Cruz); rabbit polyclonal anti-human HIF-1α (1:500, NB100-449, Novus Biologicals/R&D Systems); rat anti mouse Ter-119 (1:1000, MA1-70078, Life Technologies/Thermo Scientific); goat polyclonal anti-rat Alexa Fluor 488 (1:1000, A11006, Life Technologies/Thermo Scientific); donkey polyclonal anti-goat Alexa Fluor 488 (1:500, A-11055, Life Technologies/Thermo Scientific); rabbit polyclonal anti-goat Alexa Fluor 568 (1:1000, A-11079, Life Technologies/Thermo Scientific); donkey anti rabbit Alexa Fluor 568 (1:500, A10042, Life Technologies/Thermo Scientific); donkey anti-rabbit Alexa Fluor 350 (1:40, A10039, Life Technologies/Thermo Scientific); NeutrAvidin R-phycoerythrin (1:1000, A2660, Life Technologies/Thermo Scientific); FluoSpheres (100 nm, 580/605nm, F8801, Molecular Probes); MET inhibitor SU11274 (S1080, Selleckchem/Absource Diagnostics, Munich, Germany).

Immunoblots were performed with peroxidase-coupled secondary antibodies and enhanced chemiluminescence (ECL, Pierce/Thermo Scientific), documented with an ImageQuant LAS4000 system (GE Healthcare, Freiburg, Germany), and quantified with GelQuant.NET (version 1.8.2) provided by biochemlabsoutions.com (http://biochemlabsolutions.com).

HT1080 fibrosarcoma cells (ATCC^®^ CCL-121™, LGC Standards, Wesel, Germany) were authenticated in October 2016 using Promega PowerPlex 21 kit (Eurofins Genomics, Forensic Department, Ebersberg, Germany). Absence of mycoplasma was checked routinely (PCR Mycoplasma Detection Set, Clontech/TaKaRa, Saint-Germain-en-Laye, France). HT1080 cells were cultured in Dulbecco's modified Eagle's medium (DMEM, Gibco), supplemented with 10% fetal calf serum (FCS, Invitrogen, Karlsruhe, Germany) and penicillin/streptomycin (PAA Laboratories, Coelbe, Germany). Cells were detached with 0.5 mmol/L ethylenediaminetetraacetic acid (EDTA) in PBS without trypsin and washed with DMEM. A431 epidermoid carcinoma cells (ATCC^®^ CRL-1555™, LGC Standards, Wesel, Germany) were handled in the same way.

*NRP1*-deficient HT1080 were generated by transcription activator-like effector nuclease (TALEN) technology (TALEN Sure KO First Human, Cellectis bioresearch, Paris, France) via transient transfection with plasmids pTAL.CMVn.026484 and pTAL.CMVn.026485, targeting the first exon of NRP1 (5′-TCTGCGCCGTGCTCGCCCTCGTCCTCGCCCCG*GCCGGCGCTTTTCGCAAC*-3′; TALEN recognition site underlined, left TALEN DNA binding sequence plain, right TALEN DNA binding sequence *italicized*). TALEN mutagenesis activity was verified by deep sequencing on a GS Junior system (Roche). Both plasmids were co-transfected with pIRES-EYFP (Clontech/TaKaRa) using Fugene6 (Promega) according to the manufacturer's instructions. 24 h after transfection, green fluorescent cells were subcloned. Efficiency was monitored by T7 endonuclease (New England Biolabs, Frankfurt, Germany) assay [48] with isolated genomic DNA (QiaAmp DNA Mini and Blood Mini, Qiagen, Hilden, Germany) as template and primers *NRP1*-1ex-f 5′-CTCCTCTTTGCTGCATTTCC-3′ and *NRP1*-1ex-r 5′-GCCCAAAGACCTGAAATCCT-3′ (Eurofins Genomics, Ebersberg, Germany). For further purification of NRP1-deficient cells, hypoxia was mimicked by addition of 100 μmmol/L CoCl_2_ (Roth, Karlsruhe, Germany) to the culture medium for 24 h [49]. Cells were detached with 0.5 mmol/L EDTA in PBS and *NRP1*-expressing cells were removed with NRP1-specific magnet beads (CD304 MicroBead kit, Miltenyi, Bergisch Gladbach, Germany) and MS columns^®^ (Miltenyi) according to the manufacturer's instructions. *NRP1*-deficiency was confirmed by flow cytometry.

### Flow cytometry

For FACS analyses, cells were washed with PBS and harvested with 0.5 mmol/L EDTA. Cells were resuspended in 2 mmol/L EDTA, 2 μg/ml aprotinin in PBS and incubated 90 min with antibodies against NRP1 (1:100, rat polyclonal, Pineda, Berlin, Germany), or MET (0.2 mg/ml, sc-161, Santa Cruz, Heidelberg, Germany), or corresponding isotype controls. Subsequently, cells were washed and incubated 90 min with secondary antibodies (1:1000, donkey anti-rat Alexa Fluor 657 and goat anti-rabbit Alexa Fluor 568, both Thermo Fisher Scientific). Flow cytometry was conducted with a CyFlow cytometer and FloMax software v.2.70 (Partec, Münster, Germany).

### Real-time cell analysis

Real-time and label-free monitoring of cell migration was carried out with the xCELLigence system and RTCA software version 2.0.0.1301 (Roche, Mannheim, Germany). To this end, filter membranes of CIM plates were coated underneath with 10 μg/ml bovine collagen I in 5 mmol/L acetic acid overnight at 4°C. Rhodocetin-αβ as chemoattractant, or 10% FCS as positive control, was added to the lower compartment. 100.000 cells in DMEM, 2 mmol/L HEPES, pH 7.4, were seeded into the top compartment. SU11274 was added to both compartments. Cell migration was monitored for 24 h at 37°C in a humidified incubator at 5% CO_2_, and migration in the first two hours was quantified as Δ cell index per time unit.

### Treatment of mice with rhodocetin-αβ and FluoSpheres

All animal procedures were performed in compliance with the German Law for Welfare of Laboratory Animals and were approved by the local veterinary authority (reference number V54-19c 20/15-F146/01, regional administrative authority, Darmstadt, Germany). Tumors were generated by subcutaneous injection of 1 × 10^6^ HT1080 cells in 100 μl PBS into the right dorsomedial flank of anesthetized 6-week old female Balb/c-nu/nu nude mice (Charles River, Sulzfeld, Germany). After 21 days of tumor growth, mice had a body weight of 24 ± 5 g and were randomly divided into groups, and rhodocetin (αβγδ tetramer) at 2.5 μg/g body weight in 100 μl PBS, rhodocetin-αβ at 2 μg/g body weight in 100 μl PBS, or PBS alone as control, was injected into the tail vein. After three hours, mice were anesthetized with 1–2.5% isofluran in O_2_/compressed air (20/80, 1L/min) and analyzed by MRI. Afterwards, the deeply anesthetized mice were sacrificed by cervical dislocation and tissue samples were frozen unfixed in Tissue-Tek O.C.T. (Sakura Finetek, Staufen, Germany) on dry ice, and stored at –80°C. The number of animals per group were: *n* = 16 for rhodocetin-αβγδ-treated mice, *n* = 11 for rhodocetin-αβ-treated mice, *n* = 17 for PBS-treated control mice; *n* = 10 for rhodocetin-αβγδ-treated tumor center and rim, *n* = 5 for rhodocetin-αβ-treated tumor center and rim, *n* = 5 for PBS-treated tumor center and rim, *n* = 14 for rhodocetin-αβγδ-treated muscle, *n* = 3 for rhodocetin-αβ-treated muscle, and *n* = 14 for PBS-treated muscle as control.

Tumor-bearing mice were treated with rhodocetin-αβ as specified above. After three hours, mice were deeply anesthetized with Ketavet (Pfizer, Berlin, Germany)/Rompun (Bayer, Leverkusen, Germany) (4:1; 120μl per 25 g body weight; intraperitoneally). A suspension of 4 × 10^11^ FluoSpheres in 100 μl PBS was sonicated for 5 min and injected into the tail vein. Five minutes later, the deeply anesthetized mice were sacrificed for organ harvesting by cannulating the left ventricle with a 13G butterfly cannula and perfusion via the left ventricle at 10.8 ml/min with 40 ml ice-cold PBS followed by 40 ml 4% paraformaldehyde (PFA, Riedel-de Haën, Seelze, Germany), 0.5% glutaraldehyde (Merck, Darmstadt, Germany) in PBS. Organs were fixed for another 5 min and embedded in Tissue-Tek O.C.T., frozen on dry ice, and stored at –80°C. The number of FluoSpheres-injected animals per group were: *n* = 4 for rhodocetin-αβ-treated mice, and *n* = 3 for PBS-treated mice.

### Magnetic resonance imaging

MRI was performed using a 1.5 T scanner (Siemens Symphony, Erlangen, Germany) and a custom-made coil for radiofrequency excitation and detection. Animals were anesthetized with isoflurane (1.5%) and oxygen (0.5 l/min). For T2-weighted MRI, turbo spin echo sequence was used (orientation axial, TR 3240 ms, TE 81 ms, voxel size 0.4 × 0.4 × 1.5 mm, 3 averages, 15 images, scan time 3:40 min). For dynamic contrast-enhanced (DCE) MRI, turbo FLASH sequence was used through the largest diameter of the tumor (orientation axial, TR 13 ms, TE 5.3 ms, voxel size 0.6 × 0.6 × 2.0 mm, 240 images, scan time 14:50 min) while infusing intravenously 0.1 mmol/kg Gadomer contrast agent (Bayer-Schering Pharma, Leverkusen, Germany) over 10 s. Morphological MR images were obtained using OsiriX DICOM viewer (Bernex, Switzerland). Data from DCE-MRI was analyzed according to the pharmacokinetic two-compartment model [50] using a Dynalab workstation (Fraunhofer Mevis, Bremen, Germany) to calculate *amplitude A* ([arbitrary units], associated with relative blood volume) and *exchange rate constant k_ep_* ([1/min], reflecting vessel permeability). For DCE-MRI, regions of interest were drawn manually either around the entire tumor or separately for tumor rim and tumor center.

### Electron microscopy

Tumor-bearing mice were treated with rhodocetin-αβ or PBS as specified. For each condition two mice were analyzed. Mice were anesthetized and perfused with PBS for 1 minute and 4% PFA/PBS for 4 minutes. Kidney-, muscle-, skin-, liver- and tumor specimens were dissected and post-fixed with 4% PFA/2% glutaraldehyde in PBS overnight at 4°C. All specimens were cut into small pieces, post-fixed in 1% osmium tetroxide (8371, Roth) for two hours at room temperature and stained with 2% uranyl acetate (77870, Serva, Heidelberg, Germany) overnight at 4°C. After dehydration in graded acetone, samples were embedded in Durcupan (44611–44614, Sigma-Aldrich, Taufkirchen, Germany) and polymerized for 72 hours at 60°C. Ultrathin sections (30–50 nm) were contrast-enhanced with uranyl acetate and lead citrate (3% stabilized solution, S534/2, Leica, Wetzlar, Germany) and analyzed at 120kV on a Tecnai Spirit BioTWIN electron microscope (FEI, Eindhoven, The Netherlands) equipped with an Eagle 4k bottom-mount camera (FEI).

### Immunohistochemistry and histochemistry

Cryosections were fixed with 2% PFA in PBS for 5 minutes, blocked and permeabilized overnight at 4°C with 2% horse serum (12499C- 500ML, Sigma-Aldrich), 0.1% BSA (A1391.0500, AppliChem, Darmstadt, Germany), 0.5% saponin (S-4521, Sigma-Aldrich) in PBS, and immunostained with a CD31-specific antibody from rat (1:300, 550274, BD Pharmingen, Heidelberg, Germany), followed by a rat-specific Alexa Fluor-488-labeled secondary antibody from goat (1:1000, A11006, Life Technologies/Thermo Scientific) overnight at 4°C, respectively. Nuclei were counterstained with 20 μmol/L Hoechst 33342 (Thermo Scientific).

For immunohistochemical and histochemical double-staining, cryosections were fixed with 2% paraformaldehyde in PBS and immunostained with a CD31-specific antibody and counterstained with Hoechst 33342 as above. After image acquisition with an Eclipse Ni microscope equipped with NIS Elements software, these sections were stained with periodic acid-Schiff (PAS) stain, counterstained with hematoxylin (both Roth) according to the manufacturer's instructions and re-imaged. Micrographs were afterwards superimposed and the PAS image was given an opacity of 50% and made transparent using the ‘blend if’-slider in Adobe Photoshop CS5.

### Microscopy

Photomicrographs were acquired with an Eclipse Ni microscope equipped with NIS Elements software (v.4.30.02 build 1053, Nikon, Duesseldorf, Germany), a LSM-700 confocal microscope with ZEN 2.1 software (version 11.0.0.190, Zeiss, Oberkochen, Germany), and an Olympus IX-71 microscope equipped with a Spot-RT camera (Visitron Systems, Puchheim, Germany), and analyzed with Metamorph 7.6 software (Molecular Devices, Sunnyvale, CA, USA).

### Statistical analysis

Results were compared with GraphPad Prism (GraphPad Software, version 5.04, San Diego, CA, USA) using the unpaired two-sided t test. *P* < 0.1 was considered statistically significant.

## SUPPLEMENTARY MATERIALS FIGURES



## Abbreviations

ATV: abnormal tumor vessel
DCE-MRI: dynamic contrast-enhanced magnetic resonance imaging
DMEM: Dulbecco's modified Eagle's medium
EC: endothelial cell
EDTA: ethylenediaminetetraacetic acid
FCS: fetal calf serum
PAS: periodic acid-Schiff
PFA: paraformaldehyde
TALEN: transcription activator-like effector nuclease
TEC: tumor endothelial cell
VM: vasculogenic mimicry.
