# Supplementary material for: Rhodocetin-αβ selectively breaks the endothelial barrier of the tumor vasculature in HT1080 fibrosarcoma and A431 epidermoid carcinoma tumor models
Source: Oncotarget. 2018 Apr 27;9(32):22406–22. doi: 10.18632/oncotarget.25032 (PMC5976474; doi:10.18632/oncotarget.25032)
Supplement: Supplementary file 1 [file oncotarget-09-22406-s001.pdf]

Rhodocetin-αβ selectively breaks the endothelial barrier of the tumor vasculature in HT1080 fibrosarcoma and A431 epidermoid carcinoma tumor models

SUPPLEMENTARY MATERIALS

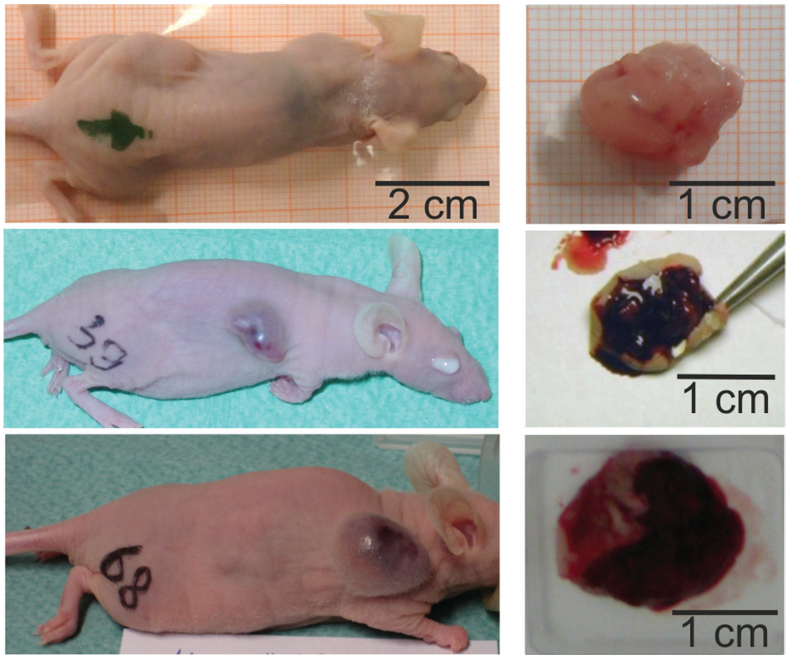

Supplementary Figure 1: Rhodocetin causes hemorrhage in solid HT1080 tumors. After 21 days of tumor growth, tumor-bearing mice were injected with either PBS, 2.5 µg/g body weight rhodocetin-αβγδ in PBS, or 2 µg/g body weight rhodocetin-αβ in PBS. Within three hours, PBS injection (top) did not show any effect, whereas rhodocetin-αβγδ (middle) and its subunit rhodocetin-αβ (bottom) caused hemorrhage exclusively in tumor tissue. Representative images are shown.

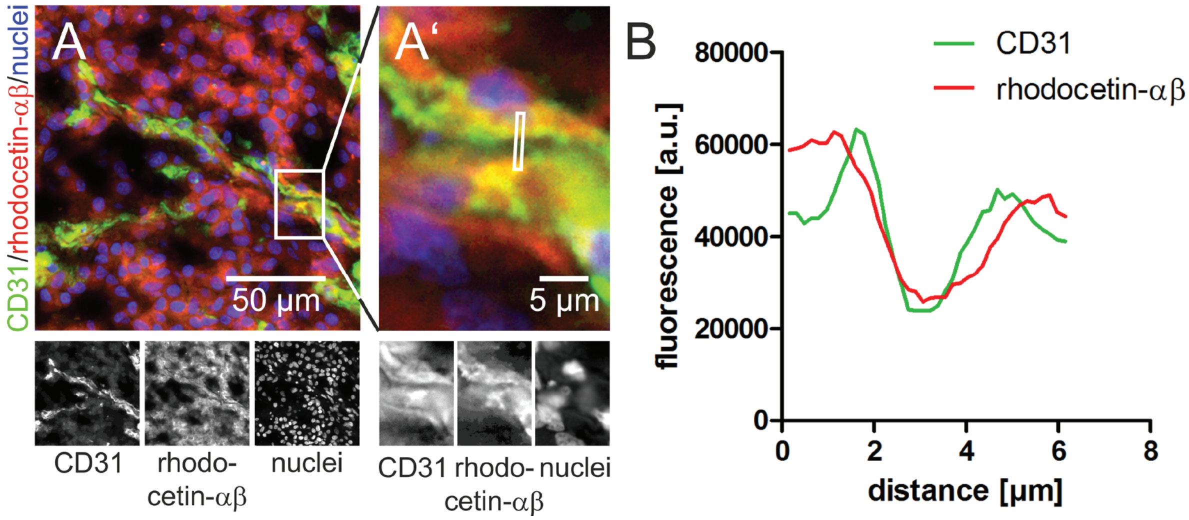

Supplementary Figure 2: Rhodocetin-αβ binds to the basolateral side of endothelial cells lining blood vessels in HT1080 tumor tissue. (**A'**) a cutout enlargement taken from Figure 1A shows a blood vessel surrounded by CD31-positive ECs (green). Intravenously injected rhodocetin-αβ (red) was detectable on the surface of HT1080 tumor cells and on $CD31^{+}$ ECs (yellow colocalization signal). (**B**) fluorescence intensity along a traceroute, averaged over a width of 5 pixels, (rectangle in **A'**) through the blood vessel revealed that in ECs rhodocetin-αβ is not bound to their apical surface but rather to their basolateral side. Original magnification was 400× (**A–A'**). Representative images are shown.

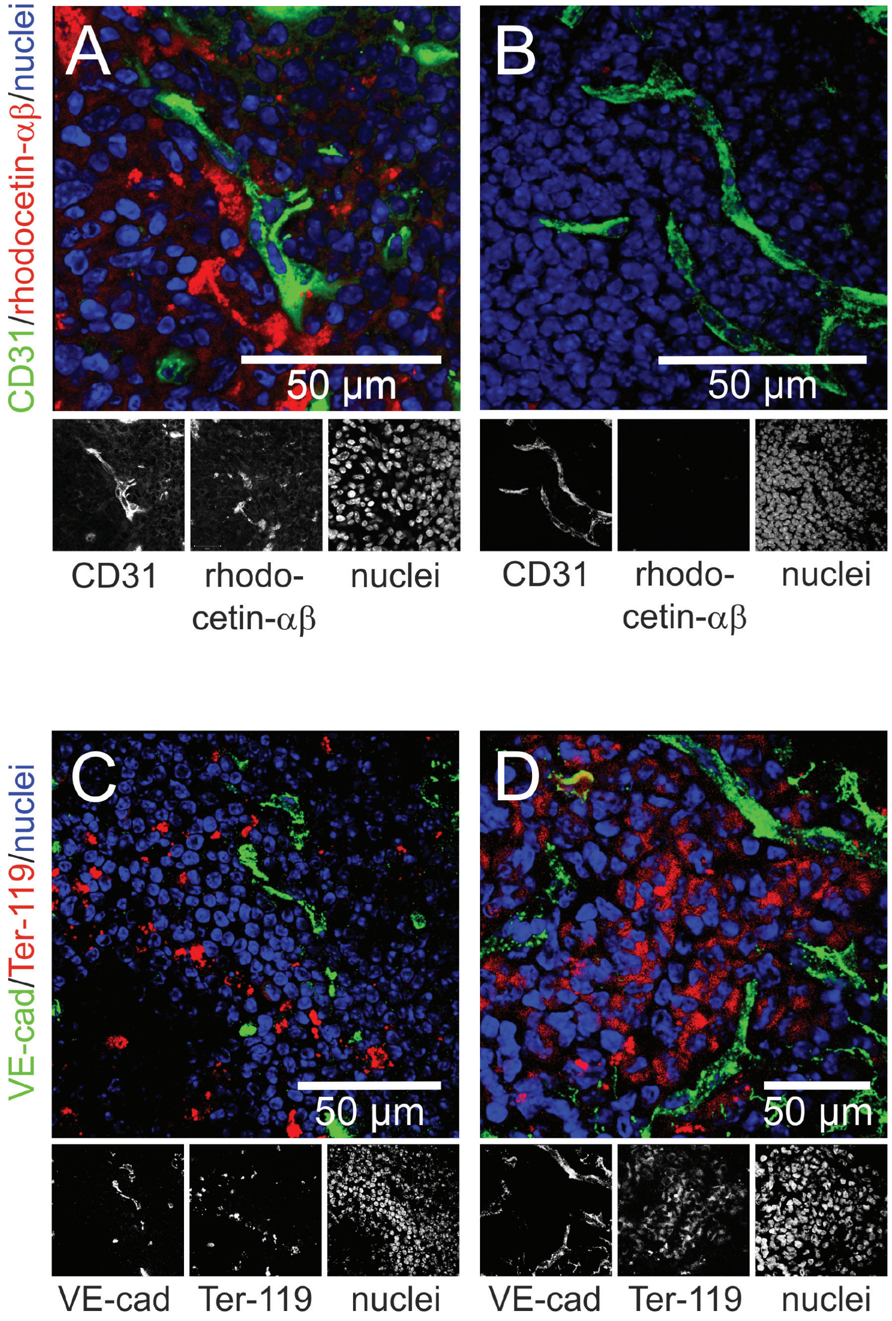

Supplementary Figure 3: Non-vascular channels in A431 tumors containing rhodocetin-αβ and blood cells. (**A**) rhodocetin-αβ was detectable in A431 tumors in reticular structures outside of blood vessels (CD31, green) with biotinylated mAb VIIF9 (red). (**B**) PBS-treated control. CD31 on ECs is labeled green, rhodocetin-αβ red, and nuclei blue. The detection of rhodocetin-αβ on endothelial and A431 tumor cells demonstrates the presence of a rhodocetin-αβ receptor. (**C**) blood cells within a VE-cadherin^{-} conduit in the A431 tumor center were detected with an antibody against the lineage marker Ter-119 (red). (**D**) rhodocetin-αβ treatment results in massive extravasation of blood cells in the A431 tumor center. VE cadherin green; Ter-119 red; nuclei blue. Original magnification was 630× (**A–D**). Representative images are shown.

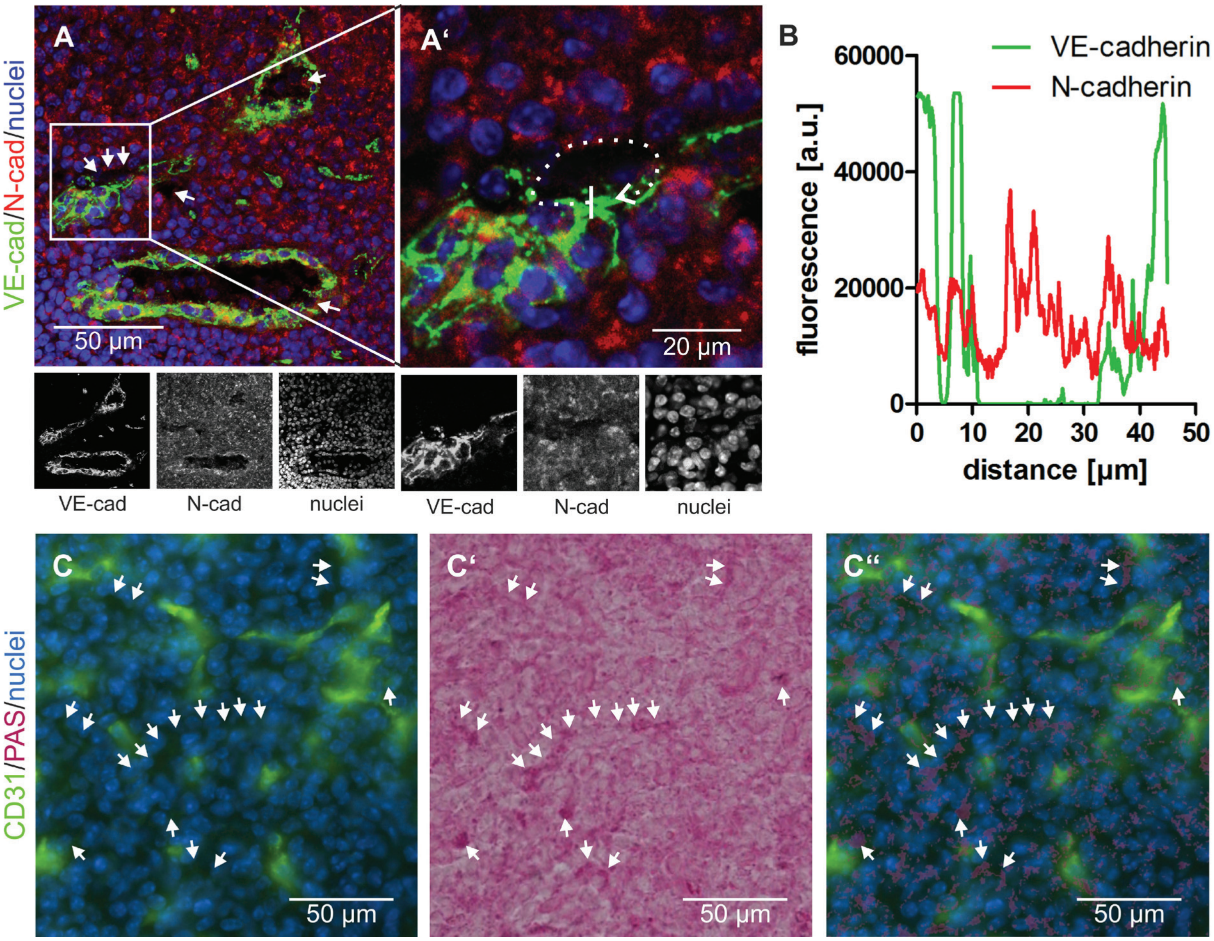

Supplementary Figure 4: Vasculogenic mimicry in A431 tumor tissue. (**A**) Abnormal tumor vessels are lined at least in part by VE-cadherin negative and N-cadherin positive tumor cells. VE-cadherin is labeled in green and N-cadherin in red. (**B**) fluorescence intensity of both signals along the dotted line marked in **A'** in clockwise direction and averaged over a width of five pixels shows the absence of the VE-cadherin signal at the top vessel wall. **C-C''**, CD31-negative/PAS-positive VM vessels were visualized in tumor tissue by consecutive immunostaining and histochemical staining of the same cryosection: Normal CD31-positive blood vessels are labeled in green (**C**, **C''**), whereas CD31-negative VM vessels are detectable by PAS staining (**C'**, **C''**). Nuclei are stained blue. Cryosections were first immunostained and photographed (**C**), subsequently histochemically PAS-stained and photographed again (**C'**), and then the images were overlaid to demonstrate numerous CD31-negative/PAS-positive VM vessels (**C''**, arrows). Original magnification was 400× (**A–A'**) and 200× (**C–C''**). Representative images are shown.

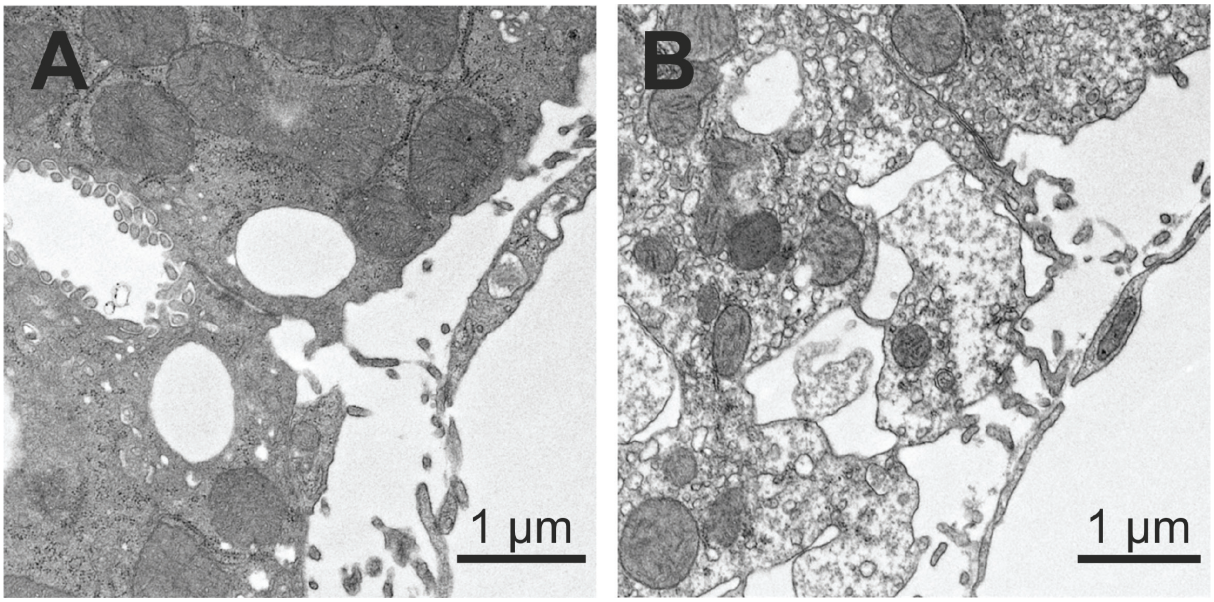

Supplementary Figure 5: Transmission electron micrographs show slight liver toxicity of rhodocetin-αβ. (**A**) PBS-treated control, (**B**) rhodocetin-αβ-treated; three hours after intravenous injection of rhodocetin-αβ. Mitochondria appear intact, but the cytoplasm shows less contrast. It is not yet clear whether rhodocetin-αβ permanently or just transiently impacts the liver. Original magnification was 6800× (**A-B**). Representative images are shown.

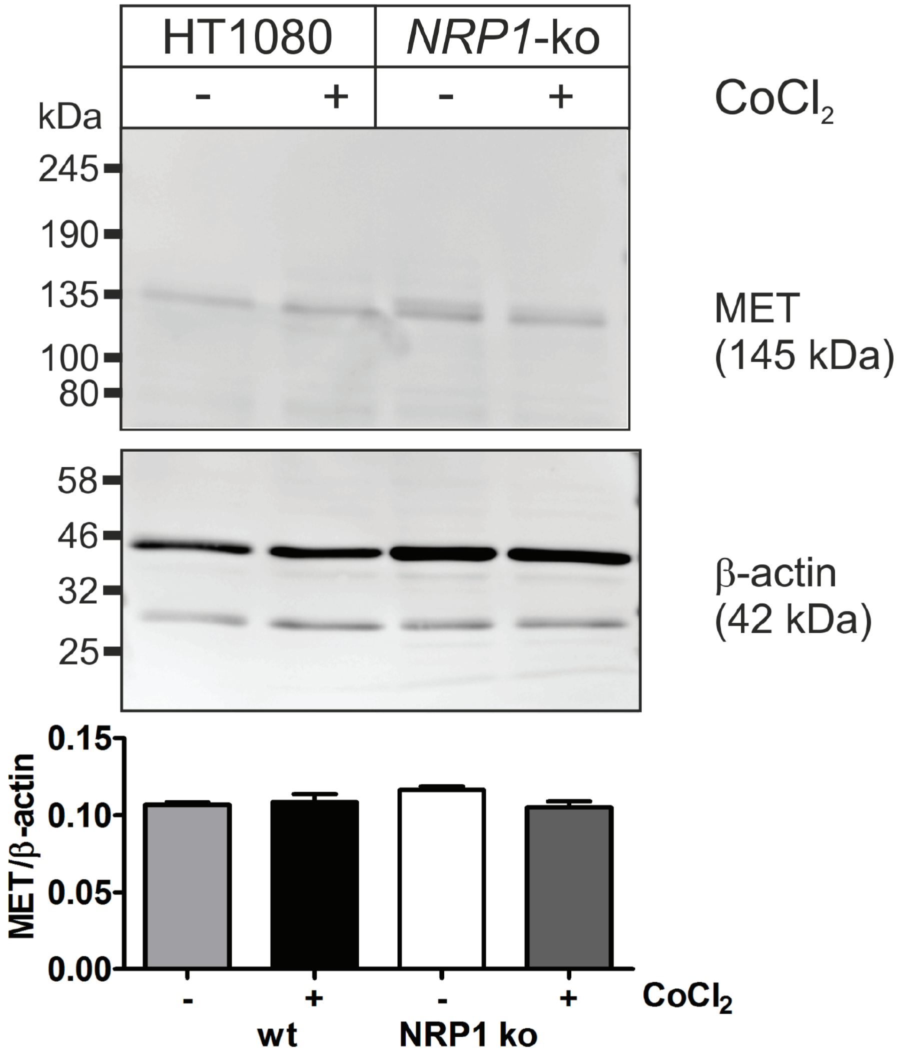

Supplementary Figure 6: MET expression by HT1080 cells is not influenced by chemically induced hypoxia. NRP1 expressing and NRP1-deficient HT1080 were treated with $CoCl_2$, mimicking a hypoxic tumor micro-environment. Immunoblotting demonstrated that their MET expression was not influenced by this treatment. The β-actin immunoblot shows even loading. Representative immunoblots are shown.

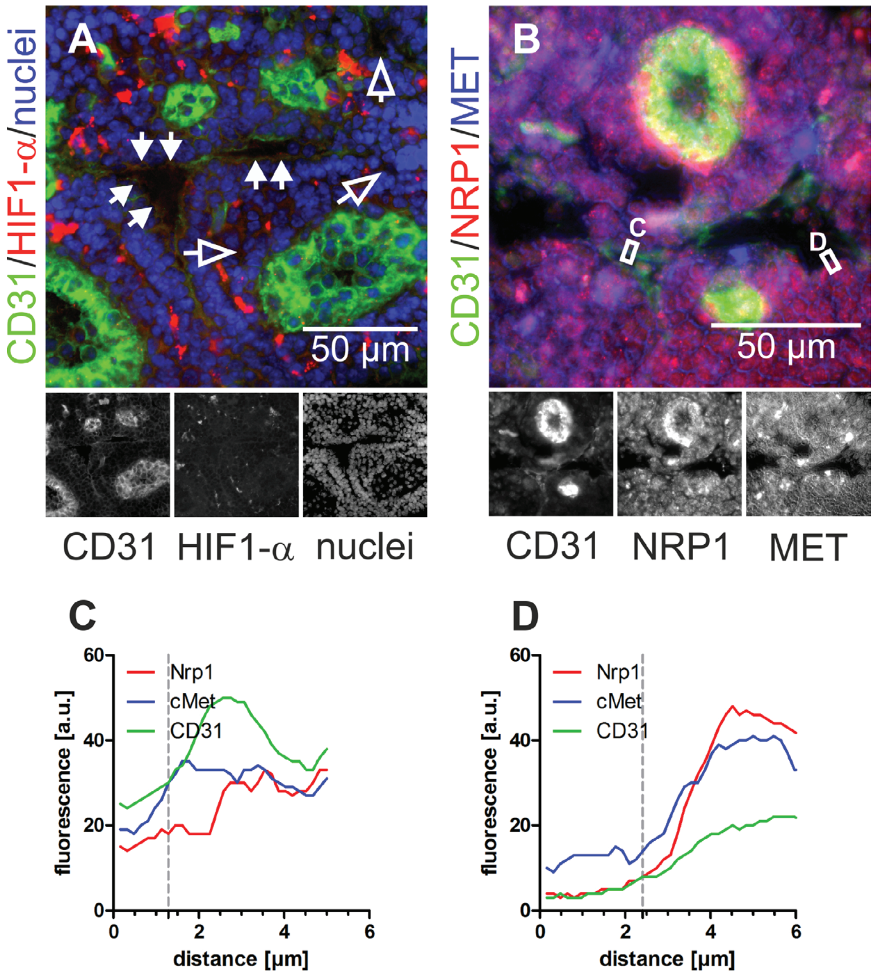

Supplementary Figure 7: In the hypoxic A431 tumor microenvironment, NRP1 and MET are present on the apical side of channel-lining A431 tumor cells in contrast to ECs. (**A**) increased HIF-1α (red) levels in hypoxic A431 tumor regions, which also contain partly (arrows) or completely (open arrows) EC-deficient VM vessels. ECs are stained green and nuclei blue. (**B**) immunostaining of NRP1 (red) and MET (blue) showed that both proteins were present on A431 cells and ECs in A431 tumor tissue. (**C**) the fluorescence intensity along a traceroute, averaged over a width of 5 pixels, (rectangle in B) through the endothelium revealed that in ECs NRP1, unlike MET, is absent from the apical side and restricted to the basolateral side. (**D)** in contrast, on ATV/VM-lining cells (rectangle in B) both NRP1 and MET are accessible from the bloodstream. Vertical gray lines in C and D indicate the position of the apical cell border. Original magnification was 400× (A–B). Representative images are shown.
